# A systematic scoping review of the multifaceted role of phoenixin in metabolism: insights from *in vitro* and *in vivo* studies

**DOI:** 10.3389/fendo.2024.1406531

**Published:** 2024-09-27

**Authors:** Adiba Najwa Muzammil, Muttiah Barathan, Muhammad Dain Yazid, Nadiah Sulaiman, Suzana Makpol, Norlinah Mohamed Ibrahim, Faizul Jaafar, Nur Atiqah Haizum Abdullah

**Affiliations:** ^1^ Department of Tissue Engineering and Regenerative Medicine, Faculty of Medicine, Universiti Kebangsaan Malaysia, Cheras, Kuala Lumpur, Malaysia; ^2^ Department of Biochemistry, Faculty of Medicine, Universiti Kebangsaan Malaysia, Cheras, Kuala Lumpur, Malaysia; ^3^ Department of Medicine, Faculty of Medicine, Universiti Kebangsaan Malaysia, Cheras, Kuala Lumpur, Malaysia; ^4^ Jeffrey Cheah School of Medicine and Health Sciences, Monash University Malaysia, Bandar Sunway, Selangor, Malaysia

**Keywords:** phoenixin (PNX), metabolism, mitochondria, glycolysis, mitochondrial respiration

## Abstract

Phoenixin (PNX) is an emerging neuropeptide that plays a significant role in regulating metabolism and reproduction. This comprehensive review examines findings from human, *in vivo*, and *in vitro* studies to elucidate the functions of PNX in metabolic processes. PNX has been identified as a key player in essential metabolic pathways, including energy homeostasis, glucose, lipid and electrolyte metabolism, and mitochondrial dynamics. It modulates food and fluid intake, influences glucose and lipid profiles, and affects mitochondrial biogenesis and function. PNX is abundantly expressed in the hypothalamus, where it plays a crucial role in regulating reproductive hormone secretion and maintaining energy balance. Furthermore, PNX is also expressed in peripheral tissues such as the heart, spleen, and pancreas, indicating its involvement in the regulation of metabolism across central and peripheral systems. PNX is a therapeutic peptide that operates through the G protein-coupled receptor 173 (GPR173) at the molecular level. It activates signaling pathways such as cAMP-protein kinase A (PKA) and Epac-ERK, which are crucial for metabolic regulation. Research suggests that PNX may be effective in managing metabolic disorders like obesity and type 2 diabetes, as well as reproductive health issues like infertility. Since metabolic processes are closely linked to reproduction, further understanding of PNX’s role in these areas is necessary to develop effective management/treatments. This review aims to highlight PNX’s involvement in metabolism and identify gaps in current knowledge regarding its impact on human health. Understanding the mechanisms of PNX’s action is crucial for the development of novel therapeutic strategies for the treatment of metabolic disorders and reproductive health issues, which are significant public health concerns globally.

## 1 Introduction 

Phoenixin (PNX) is a neuropeptide that has garnered significant interest in the scientific community over the past eight years (1). It has been identified across various species, including humans (2), rodents (3), pigs (4), cows (5), chickens (6), Xenopus (7), and zebrafish (8), highlighting its evolutionary conservation and potential biological significance.

PNX peptide derived from the C-terminal of the small integral membrane protein 20 (SMIM20) and primarily exists in two amidated isoforms: a 14-amino-acid peptide (PNX-14) and a longer, N-terminal-extended 20-amino-acid peptide (3, 9). PNX is widely distributed across various tissues, including the hypothalamus, heart, spleen, thymus, skin, ovaries, testes, adipose tissue, and pancreas (10). Notably, PNX-20 is predominantly expressed in the hypothalamus, while PNX-14 is more abundant in the heart and spinal cord (7, 11). Despite these sequence length variations, they appear to function similarly (9, 12).

PNX was characterized as a reproductive peptide or hormone upon its discovery (12). However, the specific expression levels of PNX in the brain and other tissues with respect to sex differences remain undetermined. In contrast, the well-known reproductive hormones have clearly defined expression patterns. Previous reviews have noted that reproductive hormone expression varies according to sex (13). Reproductive hormones such as gonadotrophin-releasing hormones (GnRH), luteinizing hormone (LH), follicle-stimulating hormone (FSH), progesterone, estrogen, and testosterone are present in both sexes (14). However, the expression levels and patterns of these reproductive hormones differ between sexes (15). In terms of localization, GnRH, LH and FSH were not sexually dimorphic (16, 17). However, estrogen and testosterone were localized differently in males and females (18, 19).

Previous studies have shown that the distribution of PNX in the brain is similar in both sexes (12). PNX is expressed in the arcuate nucleus (ARC) and the anteroventral periventricular nucleus (AVPV), both of which contain androgen receptors (AR) in males and females (20). Additionally, estrogen receptors (ER) are present in hypothalamic regions that express PNX, including the paraventricular nucleus (PVN), ARC, and AVPV (20–22). Furthermore, recent reviews have noted that the promoter region of PNX contains binding sites for ER (10). The presence of both AR and ER in PNX-expressing nuclei, along with ER binding sites in the promoter region, suggests that sex may influence the functional role of PNX. Further investigation into the sex-specific expression of PNX is needed to better understand its role and the associated sex-based differences.

**Figure 1:**
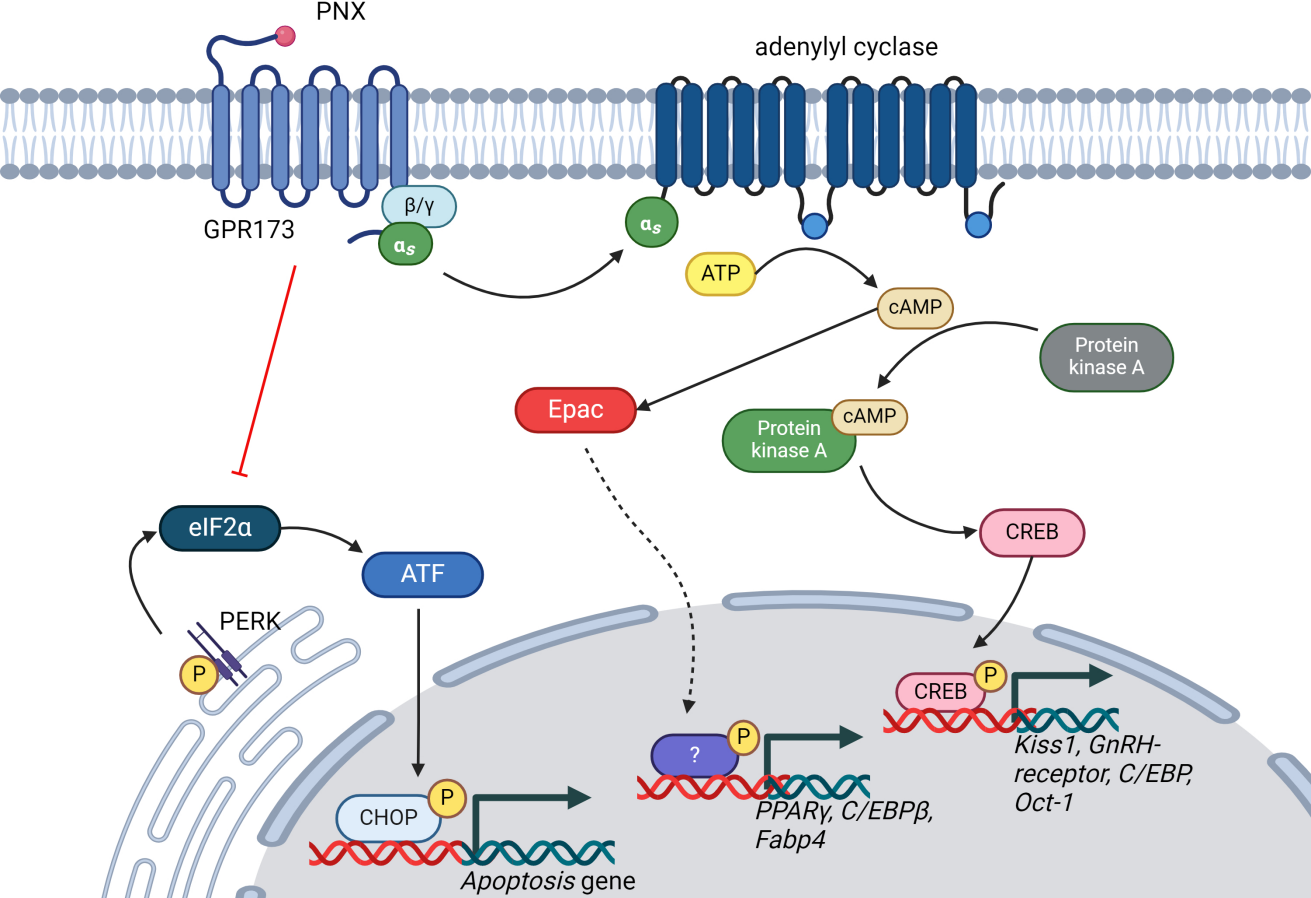
Illustration of the signaling pathways regulated by PNX. PNX has been shown to activate the cAMP/PKA and cAMP/Epac signaling pathways while inhibiting the PERK/eIF2α signaling pathway. This regulation of signaling pathways is crucial for modulating various biological processes, including reproductive systems, glucose and lipid metabolism, and apoptosis. ATP, Adenosine triphosphate; cAMP, cyclic adenosine monophosphate; CREB, cAMP-response element binding protein; GnRH, Gonadotropin hormone-releasing hormone; C/EBPs, CAAT-enhancer-binding proteins; Oct-01, Organic cation transporter 1; PPARγ, Peroxisome Proliferator-Activated Receptor Gamma; Fabp-4, Fatty Acid-Binding Proteins 4; ATF, Activating Transcription Factor; CHOP, C/EBP-homologous protein; eIF2α, Eukaryotic translation initiation factor 2α.

PNX exerts its effects primarily through the G protein-coupled receptor 173 (GPR173) (23). The binding of PNX to GPR173 is postulated to activate various intracellular signaling pathways, including the cyclic adenosine monophosphate (cAMP)/protein kinase A (PKA) (24), cAMP/exchange proteins directly activated by cAMP (Epac) (25), phosphatase and tensin homolog (PTEN)/protein kinase B (Akt) (26) and phosphoinositide 3-Kinase (PI3K)/Akt Pathway (27, 28) (**Figure 1**). Regulation of these pathways leads to downstream effects on gene and protein expression, biological processes, and physiological responses. Nonetheless, whether PNX-14 and PNX-20 differ in their bioactivity or bind to distinct receptors or receptor subtypes (24) remains uncertain.

Early findings on PNX have demonstrated its crucial roles in reproduction, particularly in regulating reproductive hormone secretion (12, 23). PNX is found to induce the stimulation of LH secretion by potentiating the activity of GnRH. At the gonadal level, PNX affects follicular development and modulates the expression of gonadotropins across various species, including fish and mammals (29). Nonetheless, recent studies have indicated that PNX is involved in a wide range of biological and physiological processes that cover anxiety (2), memory (30), oxidative stress (31), inflammation (108), cell proliferation and differentiation (33). Interestingly, recent studies have highlighted the role of PNX in regulating the biological process of metabolism (31; 34), particularly its influences on the regulation of food and fluid intake, glucose, lipid, and electrolyte metabolism, as well as mitochondrial dynamics and energy homeostasis.

It is well established that reproduction is closely interconnected to metabolic function, as metabolic health significantly influences reproductive processes (35). Various metabolic hormones, such as insulin and leptin, impact the levels of reproductive hormones and, consequently, reproductive function (36). Recent findings on PNX further strengthen the link between these two physiological functions (9). Despite significant progress in understanding the role of PNX, many questions about its involvement in metabolism remain elusive. Discussing the current findings on metabolic regulation by PNX is pivotal, as it holds the potential to unveil groundbreaking insights into its profound influence on energy regulation, metabolic homeostasis, and subsequent health outcomes (9).

Therefore, this scoping review aimed to comprehensively map the current understanding of the role of PNX in metabolism. By identifying knowledge gaps and research trends, this review paves the way for future studies to develop targeted therapeutic strategies that modulate the impact of PNX on metabolism, potentially offering novel treatment options and improving human health. These strategies could address the neuroendocrine dysregulation associated with metabolic diseases, highlighting the broader implications of PNX in maintaining metabolic homeostasis and its potential as a prognostic marker in clinical settings.

## 2 Materials and methods

This review followed the five stages outlined in the Arksey and O’Malley framework (37).

### 2.1 Identifying research questions

The following questions guided this scoping review of understanding the role of PNX in metabolism: What are the specific mechanisms underlying the involvement of PNX in energy homeostasis and metabolic regulation? What are the downstream signaling pathways activated by PNX that modulate metabolism regulation? How does PNX affect mitochondrial respiration rates and adenosine triphosphate (ATP) production in various metabolic contexts? What is the impact of PNX on glucose metabolism, insulin sensitivity, and pancreatic beta-cell function in health and metabolic disorders? How is PNX dysregulation implicated in the pathogenesis of metabolic disorders such as obesity, insulin resistance, dyslipidemia, and metabolic syndrome?

### 2.2 Identifying relevant studies

A systematic and comprehensive search strategy was employed to identify relevant studies. Four electronic databases (PubMed, Scopus, Google Scholar and Web of Science) were used to search for the articles published between 2013 and 2024. The data search was conducted on 30^th^ May 2024 using relevant keywords identified from Medical Subject Headings (MeSH). Keywords used for the data collection were PNX, metabolism, mitochondria, glycolysis and mitochondria respiration.

### 2.3 Study selection

Two independent authors screened citation titles and abstracts and then reviewed potentially relevant articles in full, with a third author responsible for resolving any arising conflicts. The screening process was carried out using Covidence. The systematic scoping review employed a stringent selection process to ensure the reliability and comprehensiveness of the included studies. Initially, articles were screened against predefined inclusion criteria, tailored to the review’s objectives. Duplicate articles were rigorously identified and removed to eliminate redundancy and maintain data accuracy. Subsequently, non-English language articles were excluded to ensure consistency and facilitate understanding. Furthermore, abstracts from conferences and symposiums were omitted to prioritize full-text articles, enhancing the depth of the review. Theses and dissertations were also excluded to focus exclusively on peer-reviewed research, emphasizing the inclusion of high-quality, published studies. Review articles and book chapters were similarly excluded to prioritize original research findings, thereby enabling a comprehensive analysis of the current knowledge landscape. By adhering to these strict exclusion criteria, the review aimed to provide a systematic and rigorous synthesis of relevant research findings, minimizing potential bias and ensuring the reliability of the conclusions drawn.

### 2.4 Data charting process

Following the application of rigorous inclusion and exclusion criteria, the selected articles underwent meticulous data extraction. A structured form was employed to gather essential details from each study, encompassing the types of experiments conducted, the specific areas of investigation, and the key findings pertaining to PNX biological function. A qualitative synthesis of the extracted data was then conducted to analyze PNX’s role in metabolism. This synthesis involved systematically evaluating and comparing the findings from the included studies, thereby offering a comprehensive assessment of the available evidence regarding PNX’s impact on metabolic processes.

### 2.5 Summarizing results

The results based on PNX’s effects on mitochondrial function, glycolytic metabolism, and mitochondrial respiration were organized under the following categories: types of PNX, action of PNX, findings, and outcomes. We reported the review following the Preferred Reporting Items for Systematic Review and Meta‐Analysis (PRISMA) guidelines –an extension for a scoping review.

## 3 Results

**Figure 2:**
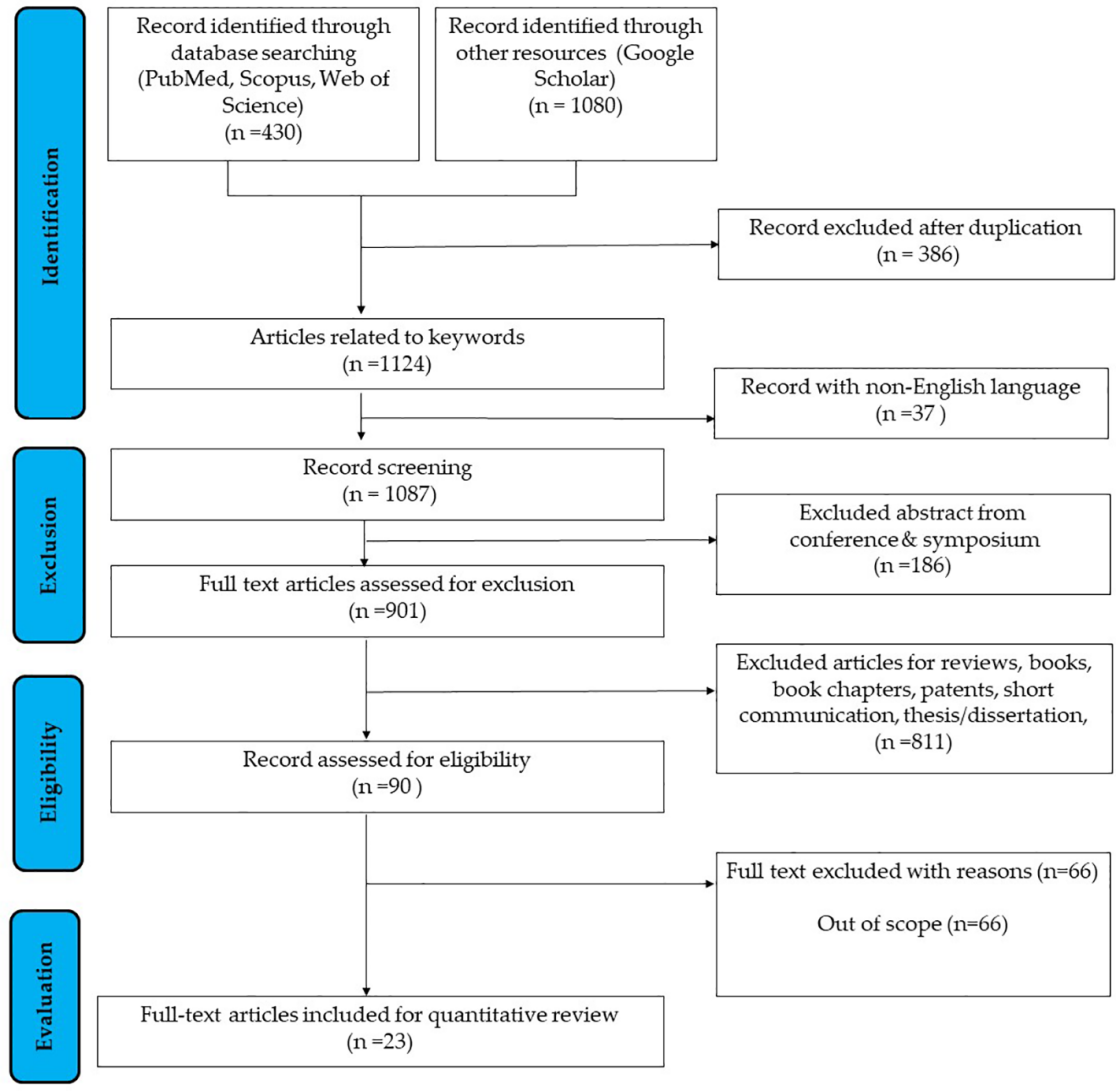
Flowchart outlining the search strategy employed in researching the correlation between PNX and metabolism (37).

The systematic review identified 23 full-text articles for comprehensive quantitative analysis from 1124 manuscripts (**Figure 2**). Based on the reviewed articles, studies on the effect of PNX on metabolism were conducted using diverse experimental designs and involved various sex-specific models or organisms. A comprehensive summary of studies on the role of PNX in metabolic regulation and its therapeutic potential is presented in **Table 1** and outlined in the following subsections.

**Table 1:** Summary of studies related to insights of PNX into metabolic regulation and therapeutic potential.

No.	Reference	Phoenixin (PNX-14 or PNX-20)	Role in Metabolism	Focus/Area of study	Human/*in vitro*/*in vivo* studies	Intervention	Findings (pathway/protein expression/intervention/mitochondria structure)	Conclusion
1.	(33)	PNX-14	Glucose metabolism	PNX’s potential to modulate insulin expression and secretion, along with the proliferation of pancreatic beta cells	*In vitro* (INS-1E cells and isolated rat pancreatic islets – Both cells isolated from male rats)	PNX-14 was administered (1, 10 and 100 nmol/l) through media to INS-1E insulin-producing cells and isolated rat pancreatic islets to evaluate its effects on insulin expression, secretion, and cell proliferation.	• PNX is present in both pancreatic alpha and beta cells. • High glucose levels stimulate the secretion of PNX from pancreatic islets. • In INS-1E cells, PNX induces the expression of *insulin* mRNA. • PNX enhances glucose-stimulated insulin secretion in both INS-1E cells and pancreatic islets. • The stimulatory effect of PNX on insulin secretion is dependent on cAMP/Epac signaling. • PNX influences cell growth and *insulin* mRNA expression through the ERK1/2 and AKT pathways.	PNX stimulates insulin expression, secretion, and INS-1E cell proliferation, suggesting its potential role in regulating glucose homeostasis through interaction with pancreatic beta cells.
2.	(38)	PNX-20	Glucose metabolism	The impact of PNX-20 on various members of the insulin-like growth factor (Igf) family in the liver and muscle of zebrafish	*In vivo* (Male and female zebrafish) and *in vitro* (zebrafish liver cells (ZFL) – The sex of the cell is unspecified)	Intraperitoneal injection of 1 ng/g, 10 ng/g, 100 ng/g, or 1,000 ng/g body weight of synthetic zebrafish PNX-20 peptide in 4 μL saline	• PNX-20 administration *in vivo* led to a decrease in *igfs, igf receptors* (*igfrs*), and *igf binding protein* (*igfbp) 5* mRNA expression in the liver of male and female zebrafish. • Incubation with PNX-20 *in vitro* resulted in the downregulation of all *igfs*, *igfrs*, and *igfbp5* mRNAs (except *igf2a*) in a zebrafish liver cell line. • PNX-20 impacted the hepatic Igf-system in zebrafish, while a noticeable sex-specific upregulation of Igf-system mRNAs was observed in the muscle.	PNX-20 acts as a tissue-specific regulator, suppressing liver Igf signaling and stimulating muscle Igf signaling in both male and female zebrafish.
3.	(39)	PNX-20	Glucose metabolism	Investigate the levels of three peptides, PNX, endocan, and spexin, in the blood and aqueous humor (AH) of patients with type 2 diabetes with and without diabetic retinopathy (DRP) and cataract	*In vivo* (Male and female human patients)	Diet, exercise, metformin (2000 mg/day), gliclazide (30 mg/day), and insulin (0.3-0.4 IU/kg/day)	• In patients with DRP and cataracts, the levels of PNX and EDC were significantly higher in both AH and blood samples compared to patients without DRP and cataracts. • Similarly, in patients with type 2 diabetes and cataracts, the levels of PNX and EDC were higher in both AH and blood samples compared to patients without diabetes and cataracts.	The potential involvement of PNX, EDC, and SPX in the development of diabetic retinopathy and cataracts in patients with type 2 diabetic
4.	(40)	PNX-14	Glucose metabolism	Investigated the effects of PNX on pancreatic injury induced by streptozotocin (STZ), nicotinamide (NAD)	*In vivo* (Adult male Sprague-Dawley rats)	Rats received saline, 0.45 nmol/kg PNX-14, or 45 nmol/kg PNX-14, and were injected intraperitoneally once daily for three consecutive days. Diabetic rats also received streptozotocin (STZ) and nicotinamide (NAD) to induce diabetes.	• PNX-14 treatment effectively prevented pancreatic damage and loss of β cells induced by STZ and NAD • PNX administration reduced oxidative stress in the pancreas, ileum, and liver tissue of diabetic rats, indicating its antioxidant properties	PNX ameliorates pancreatic injury *in vivo*.
5.	(34)	PNX-20	Glucose metabolism	The effects of PNX-20 on food intake, gene expression related to glucose transport and metabolism, and ATP production in zebrafish.	*In vivo* (Male and female zebrafish) and *in vitro* (cultured ZFL cells - The sex of the cell is unspecified)	Intraperitoneal injection of 1000 ng/g body weight of custom synthesized zebrafish PNX-20.	• PNX-20 influences feeding behavior in zebrafish, as evidenced by the reduction in food intake after PNX-20 administration. • The expression of genes involved in glucose transport and metabolism was also modulated by PNX-20, with an upregulation of glycolytic genes and a downregulation of gluconeogenic genes. • This modulation led to an increased ATP production rate associated with glycolysis in cells treated with PNX-20.	PNX-20 acts as an anorexigen in zebrafish, influencing food intake and modulating genes related to glucose metabolism.
6.	(41)	PNX-14	Glucose metabolism	Assessing the transcriptome-level changes associated with PNX exposure in female vitellogenic green-spotted pufferfish.	*In vivo* (female vitellogenic green-spotted pufferfish)	A single intramuscular injection of puffer-specific PNX-14 at a dose of 100 ng/g body weight.	• The study found that PNX-14 administration led to various responses in the hypothalamus, including proinflammatory signals and suppression of cell proliferation. • In the ovaries, PNX-14 downregulated multiple pathways and genes associated with reproductive processes, suggesting a decrease in transcriptional activity in oocytes. Both the hypothalamus and ovaries showed regulation in transforming growth factor-β and extracellular matrix remodeling pathways.	PNX appears to play important roles in regulating the immune response, energy metabolism, and cell growth
7.	(11)	PNX-14	Glucose metabolism	Investigating the expression and role of PNX in the pathogenesis of polycystic ovarian syndrome (PCOS)	*In vivo* (Female Wistar rats)	Letrozole dissolved in 2% DMSO in rapeseed oil, 1 mg/kg body weight, daily for 21 days (PCOS group).	• The expression of *Smim20* mRNA was higher in the ovary and PAT of PCOS rats, while PNX-14 peptide production was increased specifically in the ovary. • The level of the GRP173 receptor was lower in PAT but increased in the ovary of PCOS rats. The phosphorylation of kinases varied significantly depending on the tissues in PCOS rats	PNX-14 may play a role in the pathophysiology of PCOS, and it highlights novel findings regarding the mechanisms involved in PCOS development.
8.	(42)	PNX-20	Glucose and lipid metabolism	The PNX-20’s protective impact on placental insults induced by gestational diabetes mellitus (GDM)	*In vivo* (Female GDM mice)	100 ng/g/day PNX-20 administered by intraperitoneal injection for 4-6 weeks.	• GPR173 expression was identified in placental tissue. • GDM mice exhibited changes, including elevated blood glucose and lipid levels and reduced serum insulin, which these effects were reversed with PNX-20 treatment. • PNX-20 administration alleviated the excessive release of inflammatory factors and oxidative stress in GDM mice. • PNX-20 significantly suppressed the activated eIF-2α/ATF4 ER stress signaling pathway in GDM mice.	The data revealed a protective property of PNX-20 against placental insults resulted from GDM
9.	(F. 31)	PNX-14	Lipid metabolism	The effects of PNX-14 against high-fat diet (HFD)-induced NAFLD in mice	*In vivo* (HFD-induced NAFLD male mice model)	PNX-14 at a dose of 100 ng/g body weight, administered daily by gastrogavage for 10 weeks.	• PNX-14 alleviates HFD-induced obesity and fatty liver. • It reduces elevated ALT, AST, total cholesterol, and triglycerides levels. • PNX-14 increases hepatic SOD activity and GSH production while reducing MDA activity. • It decreases the production of pro-inflammatory cytokines TNF-α and IL-6. • The protective effects of PNX-14 are likely mediated through the activation of the SIRT1/AMPK and NRF2/HO-1 pathway.	PNX-14 could be a promising agent for preventing non-alcoholic fatty liver disease (NAFLD).
10.	(43)	Does not specify	Lipid metabolism	Investigating potential regulators of the neuropeptide PNX in the hypothalamus, specifically its mRNA expression levels	*In vitro* (Male and female clonal hypothalamic cell lines extracted from mouse)	Participants received treatments with compounds such as E2, BPA, sodium palmitate, sodium oleate, DHA, leptin, LPS, Forskolin, and TPA at specified concentrations and preparations.	• The study found that among the tested compounds, including hormones (estrogen and leptin), fatty acids (palmitate, docosahexaenoic acid, oleate, and palmitoleate), and the endocrine-disrupting chemical bisphenol A (BPA), only BPA and the fatty acids palmitate, DHA, and oleate were able to alter *PNX* mRNA expression levels.	PNX may play a role in sensing and responding to specific nutrients
11.	(25)	PNX-14	Lipid metabolism	The role of Phoenixin-14 (PNX) in the proliferation and differentiation of pre-adipocytes, specifically in the context of adipose tissue formation and its potential involvement in controlling body mass regulation.	*In vitro* (3T3-L1 (extracted from male mouse) and male Wistar rats used for primary preadipocyte)	The intervention was the addition of 1, 10 or 100 nmol/l of the peptide PNX-14 to the differentiation medium containing dexamethasone, IBMX and insulin, as well as to the subsequent media containing only insulin.	• PNX peptide is produced and secreted by 3T3-L1 preadipocytes and rat primary preadipocytes. • PNX promotes the proliferation and viability of 3T3-L1 preadipocytes and stimulates the expression of adipogenic genes (Pparγ, C/ebpβ, and Fabp4) in 3T3-L1 adipocytes. • The same stimulatory effects on cell proliferation and differentiation are observed in rat preadipocytes. • The mechanism of action involves the cAMP/Epac-dependent pathway, as PNX increases cAMP levels in 3T3-L1 cells and suppression of Epac signaling attenuates PNX-induced Pparγ expression without affecting cell proliferation.	Phoenixin-14 promotes the differentiation of preadipocytes into mature adipocytes, specifically white adipocytes.
12.	(44)	PNX-14 and PNX-20	Lipid metabolism	Understanding the physiological functions of two peptides, PNX14 and PNX20, generated by the small integral membrane protein 20 (SMIM20) in non-mammalian vertebrates,	*In vivo* (chicken) and *in vitro* (chicken preadipocytes) – sex unspecified	Chicken PNX14 at 10 nM and 100 nM, and cPNX20 at 10 nM and 100 nM, applied to cultured chicken primary preadipocytes during their differentiation into adipocytes, which was induced using a differentiation medium containing dexamethasone, IBMX, insulin, and oleic acid, for 2, 4, or 6 days.	• Specifically, it is found that PNX14, one of the peptides generated by *SMIM20*, plays a significant role in facilitating the differentiation of chicken preadipocytes into mature adipocytes. This effect is achieved by enhancing the expression of adipogenic genes, including *PPARγ, CEBPα*, and *FABP4*, and promoting the formation of lipid droplets. • Furthermore, the study reveals that the pro-adipogenic effect of PNX14 is mediated through the activation of the Epac-ERK signaling pathway. • This is supported by the fact that the effect is completely attenuated by the use of Epac-specific and ERK inhibitors. In contrast, cPNX20 does not exhibit a similar regulatory effect on adipogenic genes and lipid droplet content.	The signaling pathway involved in this process, involving Epac-ERK activation
13.	(45)	PNX-14, PNX-20	Lipid Metabolism	Evaluating the impact of PNX knockout (KO) on fertility, metabolic response to a high-fat diet, and behavior in mice.	C57BL/6NJ male and female mice with a whole-body knockout of the PNX	The intervention of the study involves generating and evaluating a mouse model with a whole-body knockout of the PNX gene, *Smim20.*	• PNX KO mice show normal fertility and estrous cycle lengths, indicating that the absence of PNX does not affect reproductive function. • Both wildtype and knockout mice respond similarly when placed on a high-fat diet. Nonetheless, the male heterozygous mice gain slightly less weight. • Male KO mice exhibit some changes in energy homeostasis-related genes, such as *melanocortin receptor 4* (*Mc4r*), but no changes in reproduction-related genes in the hypothalamus. • Female KO mice travel less distance in the outer zone of an open field test, suggesting alterations in anxiety or locomotor behavior.	The knockout of PNX does not significantly alter fertility or weight but modestly affects neuroendocrine genes and behavior, particularly in female mice​.
14.	(46)	PNX-20	Electrolytes metabolism	The role of the newly discovered peptide PNX in regulating vasopressin secretion and its potential effects on fluid and electrolyte homeostasis.	*In vitro* (hypothalamo-neurophysical explants – The authors do not specify the gender of the rats used.) and *in-vivo* (Male and female Sprague Dawley rats)	PNX-20 amide administered via intracerebroventricular injection at doses of 1.0 or 3.0 nmol in males, and 3.0 nmol in females.	• The PNX was shown to activate magnocellular neurons in the paraventricular nucleus (PVN) of the hypothalamus and stimulate vasopressin (AVP) release from hypothalamo-neurohypophysial explants.	PNX plays a role in controlling reproductive hormone secretion and fluid balance. It acts on the hypothalamus and pituitary gland through the Gpr173 receptor and stimulates the release of AVP.
15.	(47)	PNX-20	Electrolyte metabolism	Exploring thirst mechanisms throughout the female life cycle, and a novel mechanism has been identified which can activate ingestive behavior	*In vivo* (Male and female Sprague- Dawley rats)	The study involved various administrations into the lateral intracerebroventricular region: 1.0 or 3.0 nmol PNX-20 in 2 μL of sterile 0.9% NaCl, and 3.0 nmol PNX-20 in 2 μL of sterile 0.9% NaCl. Additionally, administrations included 2 μg (121 pmol) of siRNA targeting either eGFP or Gpr173 in 2 μL of saline on two consecutive days, and the angiotensin type 1 receptor blocker losartan (5 μg in 2 μL of vehicle) followed 20 minutes later by 3.0 nmol PNX-20 in 2 μL of vehicle.	• PNX-20 significantly increased water drinking in both male and female rats. • Reduced hypothalamic Gpr173 expression substantially decreased compensatory water intake following fluid restriction. • Increasing *PNX* and *Gpr173* mRNA levels during pregnancy suggest their potential roles in hypervolemia and heightened thirst.	PNX’s actions, including its stimulation of vasopressin secretion, suggest it may influence key neural circuits regulating ingestive behaviors and fluid/electrolyte homeostasis.
16.	(6)	PNX-14	Food intake	Investigation of the interactions between neuropeptide Y (NPY), corticotropin, melanocortin systems, and PNX-14 on food consumption in neonatal chickens.	*In vivo* (neonatal chicken) – Sex unspecified.	PNX-14 at doses of 0.8, 1.6, and 3.2 nmol was administered intracerebroventricularly (ICV).	• PNX-14 administration in neonatal chickens leads to an increase in food consumption. • Co-administration of PNX-14 with astressin-B, an antagonist of CRF1/CRF2 receptors, further enhances the hyperphagic effect of PNX-14. Co-injection of PNX-14 with astressin2-B, a CRF2 receptor antagonist, also potentiates PNX-14-induced hyperphagia. • Co-injection of PNX-14 with B5063, an NPY1 receptor antagonist, inhibits the effects of PNX-14. Co-administration of PNX-14 with SML0891, an NPY5 receptor antagonist, enhances the hypophagic effects of PNX-14	PNX-14-induced hyperphagia in neonatal chickens is mediated through the activation of NPY1, NPY5, and CRF1/CRF2 receptors.
17.	(48)	PNX-20	Food intake	The role of PNX in energy homeostasis, specifically focusing on its effects on food intake and the regulation of metabolic hormones in the gut.	*In vitro* (Mouse stomach ghrelinoma – the sex of the cells is unspecified) and *in vivo* (Male C57BL/6 J mice)	Mouse synthetic PNX-20 amide (PNX-20) at doses of 0, 1, 10, 100 and 1000 nM, administered for 2 hours to MGN3-1 cells and 6 hours to STC-1 cells.	• Presence of PNX and its receptor SREB3/GPR173 in the stomach and intestine of male mice. • Suppression of cholecystokinin (CCK) in STC-1 cells (mouse enteroendocrine cells) treated with PNX-20. • No significant effects of PNX-20 on other tested intestinal hormones (glucagon-like peptide-1, glucose-dependent insulinotropic polypeptide, and peptide YY)	PNX-20 is produced in the gut and can directly regulate the expression of metabolic hormones, particularly ghrelin and GOAT, as well as CCK.
18.	(49)	Does not specify	Food intake	Elucidating the nesfatin-1 neurons co-express PNX, and the potential physiological interplay between these two peptides in the control of crucial hypothalamus-related physiological phenomena including energy homeostasis and reproductive processes.	*In vivo* (Male Sprague–Dawley rats)	Not applicable (this is an observational study, not an intervention study)	• Distinct PNX-immunoreactivity is observed in 21–32% of cells in the arcuate nucleus, paraventricular nucleus, ventromedial hypothalamus, and lateral hypothalamus. • Nesfatin-1 expression reaches 45–68% of neurons in these sites, with co-expression notably present in 70–86% of PNX-immunoreactive neurons in the rat hypothalamic nuclei.	The widespread distribution of PNX in the hypothalamus suggests a potential functional relationship with nesfatin-1, possibly influencing the regulation of the hypothalamic-pituitary-gonadal axis or other autonomic functions.
19.	(50)	PNX-14	Food intake	Evaluating the impact of ovarian stimulation on the levels of PNX-14, NES-1, dopamine, and oxytocin, and their association with pregnancy rates.	*In vivo* (Female human)	Letrozole orally at 2.5 mg daily for 5 days, starting on day 3 of the menstrual cycle, and human chorionic gonadotropin (hCG) at 250 mcg subcutaneously, administered when at least one follicle reached ≥18 mm and endometrial thickness was ≥7 mm, to induce ovulation.	• Women who had successful pregnancies after ovarian stimulation exhibited significantly higher serum levels of PNX-14, nesfatin-1, and dopamine compared to those who did not conceive. • Elevated PNX-14 levels were strongly associated with improved pregnancy outcomes	Elevated serum levels of PNX-14, nesfatin-1, and dopamine could serve as biomarkers for predicting successful pregnancy outcomes following ovarian stimulation.
20.	(51)	PNX-14	Food intake	Determination of PNX-14 and nesfatin-1 expression patterns in the hypothalamus-pituitary-gonadal (HPG) axis during different phases of the estrous cycle.	*In vivo* (Female Wistar Albino rats)	Not applicable (the study did not involve any interventions on the participants)	• Both PNX-14 and NES-1 exhibit variable expression levels in the hypothalamus, pituitary, and gonads depending on the phase of the estrous cycle. • The fluctuating levels of PNX-14 and nesfatin-1 indicate their potential involvement in the regulation of reproductive hormone secretion and the overall function of the HPG axis.	The dynamic expression of PNX-14 and nesfatin-1 in the HPG axis during the estrous cycle highlights their importance in reproductive physiology
21.	(52)	PNX-14	Food Intake	The roles of PNX and nesfatin-1 in regulating male reproductive hormone secretion.	*In vivo* (Adult male Wistar Albino rats.	Saline (5 μl; ICV) as a control, Nesfatin-1 (200 pmol/5 μl; ICV), PNX-14 (400 pmol/5 μl; ICV), and Nesfatin-1 + PNX-14 combination (200 pmol/400 pmol in 5 μl; ICV), all administered intracerebroventricularly (ICV) in a volume of 5 μl over a period of 60 seconds.	• Both PNX and NES-1 are shown to significantly affect the secretion of key male reproductive hormones, including testosterone and luteinizing hormone (LH). • Combined treatment with both neuropeptides resulted in a more significant increase in male plasma hormone levels compared to treatment with a single neuropeptide.	Phoenixin and nesfatin-1 are important modulators of male reproductive hormones, providing new insights into their potential roles and mechanisms of action in male reproductive health.
22.	(53)	PNX-14	Mitochondrial dynamic	The potential effect of the neuropeptide PNX in obesity-induced infertility through modulating mitochondrial dynamic	*In vivo* (Adult female Albino rats)	PNX-14 administered once daily at a dose of 100 nmol/g body weight by gastrogavage for 10 weeks.	• PNX treatment in obese infertile rats leads to significant improvements in various parameters. It decreases serum levels of insulin and testosterone, as well as ovarian levels of dynamin-related protein 1, reactive oxygen species, TNF-α, MDA, and caspase-3. • PNX treatment also increases serum levels of estrogen, progesterone, LH, and FSH, along with ovarian levels of GnRH receptor, mitofusin2, mitochondrial transmembrane potential, and electron transport chain complex-I.	PNX has a role in improving obesity-induced infertility by modulating mitochondrial dynamics.
23.	(Y. 107)	PNX-20	Mitochondrial dynamics	The effects of PNX-20 on mitochondrial regulators and the underlying molecular mechanism involved.	*In vitro* (Male human neuroblastoma cell line (M17))	5 nM, 10 nM, or 20 nM of PNX-20 added to the cell culture media for 48 hours.	• The study demonstrates that PNX- 20 treatment increases the expression of mitochondrial regulators PGC-1α, NRF-1, and TFAM at both mRNA and protein levels in cultured neuronal cells. • Also shows that PNX- 20 enhances mitochondrial gene expression, mitochondrial respiratory rate, and cellular ATP production. • The activation of the CREB pathway and the presence of its tentative receptor GPR173 are involved in mediating the effects of PNX-20 on mitochondrial regulation.	PNX-20 plays a role in promoting neuronal mitochondrial biogenesis through the regulation of the CREB–PGC-1α pathway

### 3.1 Experimental designs

These 23 articles were categorized based on their experimental designs into three groups: *in vivo*, *in vitro*, and studies combining both *in vivo* and *in vitro* approaches. Of these studies, 19 were conducted using *in vivo* models, nine were conducted *in vitro*, and six were designed to investigate both *in vivo* and *in vitro*. Additionally, the review included 12 studies focusing on PNX-14, nine studies examining PNX-20, one study focusing on both PN-14 and PNX-20 and two studies that did not specify any isoform of PNX.

### 3.2 PNX and sexual differences

**Figure 3:**
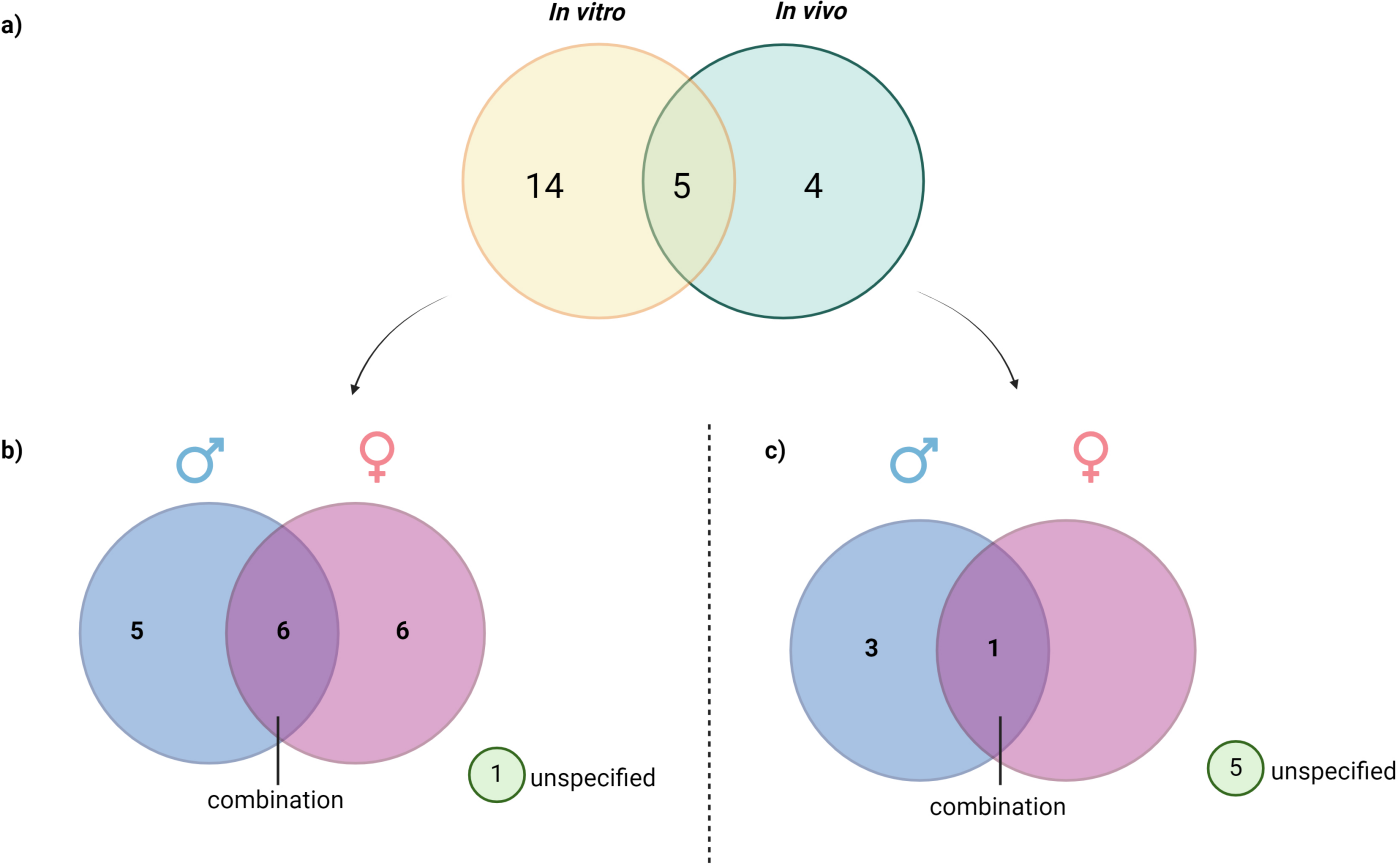
Distribution of reviewed studies by experimental type and subject sex: **(A)** Classification of reviewed studies based on the type of study, **(B)** Distribution of reviewed *in vivo* studies based on sex, **(C)** Distribution of *in vitro* study based on sex. This figure illustrates the breakdown of analyzed *in vitro* and *in vivo* studies, further categorized by the sex of subjects used (male, female, or unspecified). The visualization allows for a quick assessment of sex representation across different experimental approaches, highlighting trends in the inclusion of male, female, or sex-unspecified subjects and revealing any gaps in sex-specific research within the reviewed studies.

The full-text articles identified were categorized according to the sex of the organisms or the cell sources, including humans, rats, mice, zebrafish and pufferfish. This classification is illustrated in **Figure 3** and elicited in **Table 1**. Among the 23 studies, 15 investigated male subjects, while 14 focused on female subjects. Additionally, seven studies included both sexes to examine the role of PNX. This distribution indicates a generally balanced interest in exploring the role of PNX across sexes, with a slight preference for male subjects. However, six studies utilized *in vivo* or *in vitro* models without specifying the sex.

### 3.3 Effects of PNX-14 on metabolism

This review identified PNX-14 as playing a significant role in various metabolic processes. PNX-14 has been reported to influence both glucose and lipid metabolism, highlighting its importance in energy regulation. Additionally, since metabolic processes are closely linked with food intake, PNX-14 has been found to regulate feeding behavior, further emphasizing its involvement in maintaining metabolic balance.

#### 3.3.1 PNX-14 and glucose metabolism

PNX-14 studies have demonstrated prominent findings on the regulation of glucose metabolism. PNX-14 modulates the expression and secretion of insulin through an *in vitro* study (33). Two cells were used in this study: neuroscreen-1 (INS-1E) cells and isolated rat pancreatic islets; both cells were isolated from male rats. In this study, the treatment of PNX-14 on INS-1E insulin-producing cells induces the expression of *insulin* mRNA. Furthermore, treatment of 100 nmol/l of PNX-14 with 16.7 nmol/l of glucose on INS-1E cells and rat pancreatic islets increased insulin secretion. Apart from inducing the expression and secretion of insulin, PNX-14 was shown to increase the proliferation of INS-1E insulin-producing cells. From this study, two major signaling pathways modulated these effects in response to PNX-14 treatment (33). The PNX-14 were shown to modulate the ERK1/2 and protein kinase B (Akt) pathways that influence *insulin* mRNA expression and cell proliferation. The role of PNX-14 in glucose-stimulated insulin secretion is mediated through the cAMP/Epac signaling pathway.

The effect of PNX-14 on glucose metabolism, particularly on pancreatic tissue, was further elucidated *in vivo* setting (40). This study investigated the effects of PNX on pancreatic injury induced by streptozotocin (STZ) and nicotinamide (NAD). The PNX-14 treatment was administered intraperitoneally once daily for three consecutive days. The PNX-14 treatment significantly prevented damage and loss of β cells induced by STZ and NAD. This effect of PNX-14 in preventing cell damage and loss was postulated mediated through antioxidant properties (40). Interestingly, the PNX-14 treatment was shown to reduce the level of oxidative stress in the pancreas, ileum, and liver tissue of diabetic rats. Additionally, high-dose PNX-14 (45 nmol/kg PNX-14) treatment reduced fasting blood glucose levels in these diabetic rats without influencing plasma insulin concentrations or *insulin* mRNA expression.

Polycystic ovarian syndrome (PCOS) is a well-known metabolic disorder resulting from hyperandrogenism, ovulatory dysfunction and insulin insensitivity. The expression of PNX-14 in this metabolic disease has been studied using an animal model. This research measured the expression of PNX-14 and GPR173 in the serum, ovary and periovarian adipose tissue (PAT) in female PCOS rats compared to female control rats. The findings of this research show that the *smim-20* mRNA levels in the ovary and PAT significantly increased in PCOS rats compared to control rats. Furthermore, the serum PNX-14 levels were significantly increased in PCOS rats (11). In addition, the alteration of PNX-14 and GPR173 expression was associated with the changes in the PI3K/Akt signaling pathway (11), postulating the possible role of PNX-14 in the pathophysiology of PCOS.

The effect of PNX-14 *in vivo* was also further elucidated using fish or non-mammal animal models. This study used female vitellogenic green-spotted pufferfish. The puffer-specific PNX-14 was administered through a single intramuscular injection at a dose of 100 ng/g body weight. The transcriptomic analysis was performed in the hypothalamus and ovary. The findings demonstrated that the PNX-14 treatment changes the gene sets related to immune responses, including cytokine signaling and natural killer cell activation in both the hypothalamus and ovary. Interestingly, the findings show that PNX-14 modulates the diabetes-related and glucose metabolism gene expression in the hypothalamus alone. The study suggested that PNX-14 plays an important role in regulating the immune response and energy metabolism.

#### 3.3.2 PNX-14 and lipid metabolism

Apart from glucose metabolism, PNX-14 shows a prominent role in modulating lipid metabolism in several studies. Firstly, the role of PNX-20 was determined using high-fat diet (HFD)-induced non-fatty acid liver disease (NAFLD) male mice (31). The PNX-20 was administered daily by gastrogavage for 10 weeks at a dose of 100 ng/g body weight. The findings of this study show that PNX-14 reduces elevated levels of ALT, AST, total cholesterol, and triglycerides in HFD-induced obesity and fatty liver mice. In addition, PNX-14 increases hepatic SOD activity and GSH production while reducing MDA activity. The protective effects of PNX-14 are likely mediated through the activation of the sirtuin 1 (SIRT1)/AMP-activated protein kinase (AMPK) and Nuclear respiratory factor 2 (NRF2)/heme oxygenase 1 (HO-1) pathway. From this study, PNX-14 has been postulated to have protective effects against high-fat diet-induced non-alcoholic fatty liver disease (NAFLD) in mice (31).

Another study of PNX-14 shows its role in pre-adipocyte proliferation and differentiation (25). This study used an *in vitro* approach that utilized the use of 3T3-L1 cells (extracted from male mice) and primary preadipocytes (extracted from male Wistar rats). The PNX-14 treatment using 1, 10 or 100 nmol/l of the peptide PNX-14 to the differentiation medium containing dexamethasone, IBMX and insulin, as well as to the subsequent media containing only insulin. There are several key findings from this study. Firstly, the PNX peptide is produced and secreted by 3T3-L1 preadipocytes and rat primary preadipocytes. Secondly, the PNX-14 treatment promotes the proliferation and viability of 3T3-L1 preadipocytes and stimulates the expression of adipogenic genes (*Pparγ*, *C/ebpβ*, and *Fabp4*) in 3T3-L1 adipocytes. Furthermore, the same stimulatory effects on cell proliferation and differentiation are observed in rat preadipocytes with PNX-14 treatment. This study shows the differentiation of preadipocytes into mature adipocytes, particularly white adipocytes, was through the cAMP/Epac-dependent pathway (25). Therefore, PNX-14 is postulated to promote the differentiation of preadipocytes into mature adipocytes, specifically white adipocytes.

The role of PNX-14 in lipid metabolism is further studied in non-mammalian vertebrate settings. This study utilized an *in vitro* study in which they used chicken preadipocytes (44). The chicken PNX-14 was used to treat the preadipocytes at 10 nM and 100 nM. The finding shows that the cPNX-14 enhanced the expression of adipogenic genes, including *PPARγ*, *CEBPα*, and *FABP4*, and promoted the formation of lipid droplets. Furthermore, the study reveals that the pro-adipogenic effect of PNX14 is mediated through the activation of the Epac-ERK signaling pathway. The similarity of findings with the mammalian animal model highlights PNX evolutionary conservation and potential as a therapeutic target across species (44).

Despite using the intervention by treating *in vivo* and *in vitro* with PNX-14, the knockout method has also been utilized to understand the role of PNX-14. A recent study utilized C57BL/6NJ male and female mice with a whole-body knockout of the PNX to evaluate the impact of PNX knockout (KO) on fertility, metabolic response to a high-fat diet, and behavior in mice (45). The findings of this study show that PNX KO mice show normal fertility and estrous cycle lengths. Furthermore, the male PNX KO mice exhibit some changes in energy homeostasis-related genes, such as *melanocortin receptor 4* (*Mc4r*), but no changes in reproduction-related genes in the hypothalamus (45). In addition, both wildtype and knockout mice respond similarly when placed on a high-fat diet. Nonetheless, the male heterozygous mice gain slightly less weight. Meanwhile, female PNX KO mice travel less distance in the outer zone of an open field test, suggesting alterations in anxiety or locomotor behavior. Therefore, this study postulated that the knockout of PNX does not significantly alter fertility or weight but modestly affects neuroendocrine genes and behavior, particularly in female mice.

#### 3.3.3 PNX-14 and food intake regulation

The role of PNX-14 in food intake has garnered significant attention from the researcher. Several studies have been conducted to evaluate the potential role of PNX-14 in food intake regulation. A recent study was conducted to determine the impact of ovarian stimulation on the levels of PNX-14, NES-1, dopamine, and oxytocin and their association with pregnancy rates among pregnant women (50). This prospective case-control study found that elevated levels of the neuropeptides PNX-14, nesfatin-1 (NES-1), and dopamine, along with decreased oxytocin (OT) levels, were associated with positive pregnancy rates in infertile women after ovarian stimulation (50). From this study, PNX-14, nesfatin-1, and dopamine are postulated to serve as potential biomarkers for predicting successful pregnancy outcomes following ovarian stimulation.

The findings from the prospective case-control study mentioned before are consistent with the findings using an *in vivo* setting. In this study, female Wistar Albino rats were used to determine the PNX-14 and nesfatin-1 expression patterns in the hypothalamus-pituitary-gonadal (HPG) axis during different phases of the estrous cycle (51). The findings of this study show that both PNX-14 and nesfatin-1 exhibit variable expression levels in the hypothalamus, pituitary, and gonads, depending on the phase of the estrous cycle. Therefore, it is postulated that the fluctuating levels of PNX-14 and nesfatin-1 indicate their potential involvement in regulating reproductive hormone secretion and the overall function of the HPG axis. In addition, the co-expression of PNX-14 and NES-1 shows an association between a reproductive system and energy homeostasis through food intake regulation (51).

Further study on the PNX-14 effect on food regulation was conducted using adult male Wistar Albino rats (52). This study determined the role of PNX-14 and NES-1 in regulating male reproductive hormone secretion. This study used saline as a control, NES-1 (200 pmol), PNX-14 (400 pmol), and NES-1 + PNX-14 combination (200 pmol/400 pmol) as treatment group. All compounds were administered intracerebroventricularly (ICV) in a volume of 5 μl for 60 seconds. The findings of this study show that both PNX-14 and NES-1 are shown to significantly affect the secretion of key male reproductive hormones, including testosterone and LH. Interestingly, combined treatment with both neuropeptides resulted in a more significant increase in male plasma hormone levels than treatment with a single neuropeptide. Therefore, the findings from this study postulated that PNX-14 and NES-1 are modulators of male reproductive hormones, providing new insights into their potential in male reproductive health and food intake.

The role of PNX-14 was also elucidated using an intervention setting in an *in vivo* setting. A study of chickens has shown that intracerebroventricular (ICV) injections of PNX-14 increased food consumption in neonatal chickens (6). Co-administration of PNX-14 with Astressin-B, an antagonist of CRF1/CRF2 receptors, further enhances the hyperphagic effect of PNX-14. In addition, the co-injection of PNX-14 with astressin2-B, a CRF2 receptor antagonist, also potentiates PNX-14-induced hyperphagia. Meanwhile, co-injection of PNX-14 with B5063, an NPY1 receptor antagonist, inhibits the effects of PNX-14. Lastly, co-administration of PNX-14 with SML0891, an NPY5 receptor antagonist, enhances the hypophagia effects of PNX-14. From these findings, it is postulated that PNX-14-induced hyperphagia in neonatal chickens. Furthermore, this study suggested that the hyperphagia activation is mediated by activating NPY1, NPY5, and CRF1/CRF2 receptors.

### 3.4 Effects of PNX-20 on metabolism

Similarly, PNX-20 is implicated in the regulation of glucose and lipid metabolism, as well as food intake. However, this review reveals that PNX-20 has additional roles beyond those shared with PNX-14. Specifically, PNX-20 has been reported to influence electrolyte metabolism and mitochondrial dynamics—functions not attributed to PNX-14. This suggests that while both PNXs share some common metabolic roles, PNX-20 may have broader effects on metabolic processes.

#### 3.4.1 PNX-20 and glucose metabolism

Besides PNX-14, studies on PNX-20 reveal its role in glucose metabolism. Firstly, the animal study has shown that the PNX-20 treatment ameliorated the symptoms of gestational diabetes mellitus (GDM) (42). This study used female GDM mice, and the PNX-20 treatment was administered through intraperitoneal injection for 4-6 weeks. From this study, the PNX receptor, GPR173, was identified in placental tissue. Furthermore, PNX-20 treatment has been shown to reverse elevated blood glucose and lipid levels and reduce serum insulin exhibited in GDM mice. In addition, PNX-20 treatment alleviated the excessive release of inflammatory factors and oxidative stress in GDM mice. In terms of signaling pathways, PNX-20 significantly suppressed the activated eIF-2α/ATF4 ER stress signaling pathway in GDM mice. Therefore, this study postulated that PNX-20 has protective properties against placental insults resulting from GDM.

Interestingly, the role of PNX-20 in glucose metabolism was further studied in the non-vertebrate study (38). This study used *in vivo* (male and female zebrafish) and *in vitro* (cultured ZFL cells - The sex of the cell is unspecified). The custom synthesized zebrafish PNX-20 was administered intraperitoneal injection at 1000 ng/g body weight. The finding of this study shows that PNX-20 significantly downregulated the mRNA expression of *insulin growth factor (IGFs)*, *IGF receptors (IGFRs)*, and *IGF binding protein 5 (igfbp5)* in the liver of both male and female zebrafish (38). A contradicting finding was observed in the muscle where PNX-20 treatment significantly upregulated the mRNA expression of *IGFs* and *IGF receptors* (38). Furthermore, PNX-20 treatment in zebrafish significantly increased the mRNA expression of *glycolytic* genes and decreased the *gluconeogenic* genes (34). In addition, a higher ATP production rate from glycolysis was observed with PNX-20 treatment. Thus, this study postulated that PNX-20 acts as an anorexigenic molecule in zebrafish, influencing food intake and modulating genes related to glucose metabolism.

#### 3.4.2 PNX-20 and lipid metabolism

Similar to PNX-14, PNX-20 is prominent in modulating lipid metabolism in several studies. Firstly, the *in vivo* study used female GDM mice to determine the effect of PNX-20 in lipid metabolism. The PNX-20 treatment was administered through intraperitoneal injection for 4-6 weeks at 100 ng/g/day. The finding from this study found that PNX-20 improved the lipid profile, including the total serum cholesterol (TCH), serum triglyceride (TG), high-density lipoprotein (HDL) and low-density lipoprotein (LDL). Therefore, this study postulated that PNX-20 plays a significant role in regulating lipid metabolism, particularly in terms of cholesterol and lipoprotein levels.

A contrary finding on the effect PNX-20 in lipid metabolism was observed in chickens (44). This study utilized an *in vitro* study in which they used chicken preadipocytes. In this study, both PNX-14 and PNX-20 were used as treatment. In addition, the effect of these two PNX isomers were compared. The findings from this study show that the treatment of PNX-20 did not affect the expression of adipogenic genes and lipid droplet content (44). This study postulated that the PNX-20 is not involved in the regulation of lipid metabolism in chickens.

Besides the intervention study, a recent study has demonstrated that male KO (PNX-14 & 20) mice exhibited changes in energy homeostasis-related genes, such as *melanocortin receptor 4* (*Mc4r*), but did not show alterations in reproduction-related genes in the hypothalamus (45). From this study, it is postulated that the knockout of PNX, which includes PNX-20, resulted in changes in the regulation of lipid metabolism, particularly in the regulation of obesity.

#### 3.4.3 PNX-20 and electrolyte metabolism

From the reviewed articles, the effect of PNX on electrolyte metabolism is only studied using the PNX-20 isoform. Firstly, a recent study used male and female Sprague-Dawley rats to elucidate thirst mechanisms throughout the female life cycle, and a novel mechanism has been identified which can activate ingestive behavior (47). The PNX-20 was administrations into the lateral intracerebroventricular region at the dosage of 1.0 or 3.0 nmol PNX-20 This study demonstrated that PNX-20 increases water intake in both male and female rats, suggesting its involvement in hypervolemia and heightened thirst during pregnancy (47). Therefore, it is postulated that PNX-20 may influence key neural circuits regulating ingestive behaviors and fluid/electrolyte homeostasis.

Another study used *in vitro* (hypothalamic-neurophysical explants –sex unspecified) and *in vivo* (male and female Sprague Dawley rats) to elucidate the role of PNX-20 in regulating vasopressin secretion and its potential effects on fluid and electrolyte homeostasis (46). The PNX-20 was administered via intracerebroventricular injection at doses of 1.0 or 3.0 nmol in males and 3.0 nmol in females. The findings of this study show that PNX-20 stimulates vasopressin release from hypothalamic-neurohypophysial explants, indicating its role in controlling fluid and electrolyte homeostasis (46). Therefore, this study postulated that PNX-20 plays a role in controlling reproductive hormone secretion and fluid balance.

#### 3.4.4 PNX-20 and food intake

Besides fluid intake regulation, PNX-20 regulates the expression of metabolic hormones that are involved in regulating food intake. Only one study elucidates the role of PNX in energy homeostasis, specifically focusing on its effects on food intake and the regulation of metabolic hormones in the gut (48). This study used *in vitro* (MGN3-1 cells and STC-1 cells, mouse stomach ghrelinoma – the sex of the cells is unspecified) and *in vivo* (male C57BL/6 J mice). The *in vivo* is meant to determine the expression level of PNX-20 in the stomach and intestine. The mouse synthetic PNX-20 amide (PNX-20) was used to treat both cells at doses of 0, 1, 10, 100 and 1000 nM for 2 hours to MGN3-1 cells and 6 hours to STC-1 cells. The findings of this study show that PNX-20 and its receptor SREB3/GPR173 is expressed in the stomach and intestine of male mice. Furthermore, the PNX-20 upregulate the mRNA expression of ghrelin and ghrelin O-acyltransferase (GOAT) and suppresses the mRNA expression of cholecystokinin (CCK) in STC-1 cells (48). This study postulated that PNX-20 directly regulates the expression of metabolic hormones, particularly ghrelin, GOAT and CCK.

#### 3.4.5 PNX-20 and mitochondrial dynamics

Apart from PNX-14, PNX-20 is also involved in regulating the mitochondrial dynamic. A recent study elucidates the effects of PNX-20 on mitochondrial regulators and the underlying molecular mechanism involved (107). This study used *in vitro* [male human neuroblastoma cell line (M17)] and PNX-20 at dosages of 5 nM, 10 nM, or 20 nM as a treatment for this cell. The findings of this study show that PNX- 20 treatment increases the expression of mitochondrial regulators PGC-1α, NRF-1, and TFAM at both mRNA and protein levels in cultured neuronal cells. Furthermore, PNX-20 enhances mitochondrial gene expression, mitochondrial respiratory rate, and cellular ATP production. In addition, the activation of the CREB pathway, along with the PNX receptor, GPR173, plays a role in mediating the effects of PNX-20 on mitochondrial regulation. From these findings, this study postulated that PNX-20 plays a role in promoting neuronal mitochondrial biogenesis by regulating the CREB–PGC-1α pathway.

### 3.5 Effects of PNX (unspecified) on metabolism

Three studies did not specify whether PNX-14 or PNX-20 was being measured. In these articles, the authors generally refer to the compound as PNX. Nevertheless, these studies highlighted the findings on the impact of PNX on glucose and lipid metabolism and food intake.

#### 3.5.1 PNX and glucose metabolism

A recent study investigates the levels of three peptides, PNX, endocan, and spexin, in the blood and aqueous humor (AH) of patients with type 2 diabetes with and without diabetic retinopathy (DRP) and cataract (39). This study recruited male and female patients, and the PNX level was measured. The investigation of PNX in the blood and aqueous humor of patients with type 2 diabetes with and without diabetic retinopathy and cataracts has found interesting findings. The levels of PNX were significantly higher in the aqueous humor and blood of patients with diabetic retinopathy and cataracts and patients with diabetes mellitus and cataracts compared to patients with only cataracts (39). This study postulated that PNX is involved in the development of diabetic retinopathy and cataracts in patients with type 2 diabetes.

#### 3.5.2 PNX and lipid metabolism

Apart from glucose metabolism, a non-intervention study investigates the potential regulators of the *PNX* mRNA expression in the hypothalamus (43). This *in vitro* study used male and female clonal hypothalamic cell lines extracted from mice. This has shown that the expression of PNX was upregulated by the fatty acids palmitate, DHA, and oleate, suggesting a role for PNX in lipid metabolism and nutritional sensing (43). Thus, this study postulated that PNX may play a role in sensing and responding to specific nutrients.

#### 3.5.3 PNX and food intake

A previous study elucidates the potential physiological interplay between PNX and NES-1 peptides in controlling crucial hypothalamus-related physiological phenomena, including energy homeostasis and reproductive processes (49). This is an observational study that used male Sprague–Dawley rats. The findings have shown that NES-1 and PNX have a similar distribution pattern in the rat hypothalamus (49). These findings suggest that PNX has a potential functional relationship with NES-1, possibly influencing the hypothalamic-pituitary-gonadal axis regulation or other autonomic functions.

## 4 Discussion

This systematic scoping review of 23 studies highlights the multifaceted roles of PNX peptides in metabolic regulation, drawing on a wide range of human, *in vivo* (vertebrate and non-vertebrate) and *in vitro* studies. The findings indicate that phoenixin significantly influences complex metabolic regulation, including energy homeostasis, food and fluid intake, glucose and lipid metabolism, and mitochondrial dynamics. Moreover, the review highlights the interplay between metabolic functions and reproductive health of PNX, particularly in conditions such as polycystic ovary syndrome (PCOS). These findings suggest that phoenixin is a pivotal regulatory peptide with substantial implications for both metabolic and reproductive health, warranting further research into its underlying mechanisms and therapeutic potential.

### 4.1 The Expression of PNX

**Figure 4:**
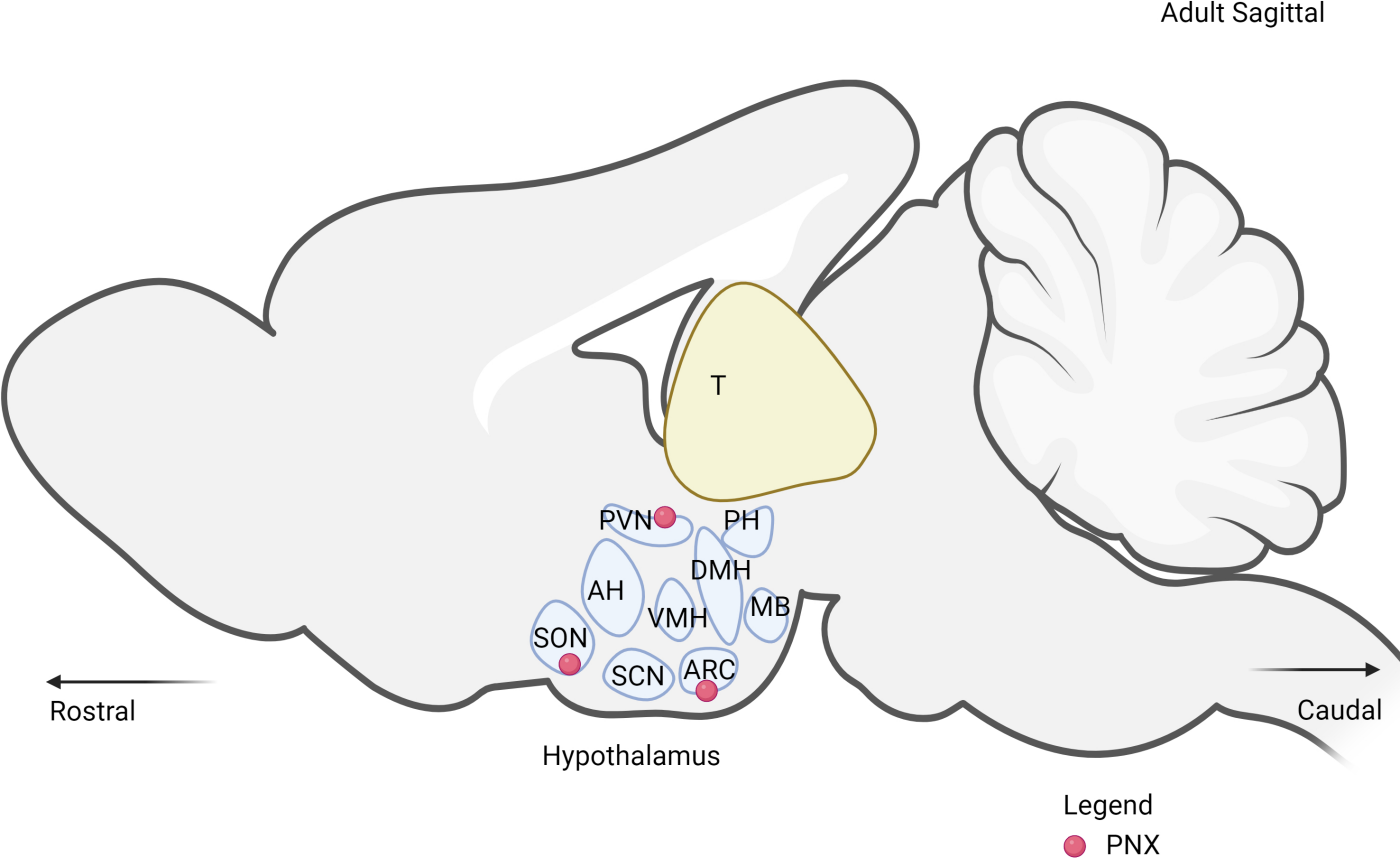
The expression of PNX in the hypothalamus. PVN, paraventricular nucleus; SON, supraoptic nucleus; ARC, arcuate nucleus; DMH, dorsomedial hypothalamic nucleus; VMH, ventromedial hypothalamus; SCN, suprachiasmatic nucleus; MB, mammillary bodies; PH, posterior hypothalamus.

It is well documented that PNX-20 is mainly expressed in the central nervous system (CNS), particularly in the hypothalamus, and PNX-14 is expressed in the peripheral system. In the hypothalamus, PNX is highly expressed in key regions such as the paraventricular nucleus (PVN), supraoptic nucleus (SON), arcuate nucleus (ARC), and anteroventral periventricular nucleus (AVPV) (**Figure 4**) (10). Out of these regions, ARC and PVN are primarily responsible for the regulation of metabolism (54, 55). Within the ARC, PNX has been observed to co-express with various neuropeptides such as NES-1 (51), neuropeptide Y (NPY) (56), and agouti-related peptide (AgRP) (57). These peptides collectively play essential roles in the complex signals network regulating hunger, energy expenditure, and overall metabolic processes. In the PVN, several key hormones and neuropeptides that are responsible for metabolism are expressed in these nuclei, including corticotropin-releasing hormone (CRH), thyrotropin-releasing hormone (TRH) and oxytocin (OT) (58). The expression of PNX in both nuclei indicates the presence of interaction with each other to regulate metabolic processes through both synergistic and antagonistic effects. This phenomenon has been observed with the reproductive neuropeptide kisspeptin, which seems to directly interact with both NPY/AgRP and POMC/CART neurons in a bidirectional manner (59). 

Beyond the CNS, PNX is expressed in various peripheral organs involved in metabolic regulation, including the heart, pancreas, and adipose tissue. The expression of PNX in the heart is implicated in cardiovascular regulation by modulating heart contractility and relaxation in normal and obese rats (60). Proper cardiac function is essential for maintaining optimal blood flow and nutrient delivery to tissues, influencing overall metabolic efficiency (61). In the pancreas, the expression of PNX influences insulin secretion (33). Furthermore, PNX has been found to alleviate pancreatic injury induced by streptozotocin (STZ) and nicotinamide (NAD) in diabetic rats (40). Lastly, the presence of PNX in adipose tissue promotes the differentiation of preadipocytes into mature adipocytes through pathways such as cAMP/Epac and Epac-ERK, enhancing adipogenesis and playing a critical role in lipid storage and utilization (25). The dual expression of PNX in both the hypothalamus and peripheral organs highlights its integrative role in coordinating metabolic functions.

Apart from the expression of PNX, the reviewed studies demonstrate a significant interest in the sex-specific effects of PNX on metabolic regulation. Among the 23 studies examined, the distribution suggests a relatively balanced interest in understanding the role of PNX across sexes, though there is a slight emphasis on male subjects. Both phoenixin and nesfatin-1 significantly increased the secretion of follicle-stimulating hormone (FSH), LH, and testosterone in rat plasma without inducing any changes in plasma GnRH, thus indicating that both these neuropeptides play a synergistic role in regulating male sex hormones (52). Furthermore, a previous study has shown that PNX modulates the expression of estrogen and progesterone in obesity-induced female rats (53). These findings emphasized the integration between reproduction and metabolism regulation modulation through PNX in both sexes.

Interestingly, one study has indicated that PNX treatment exerts different mechanisms of action in different sexes (34). This study demonstrates that PNX treatment upregulates the expression of ghrelin O-acyl-transferase (GOAT), peptide YY (PYY) and cholecystokinin (CCK), which only can be observed in female fish (34). All these proteins play a significant role in regulating appetite, digestion, and overall metabolic processes (32, 62, 63). For instance, a previous study demonstrated that estrogen increases CCK mRNA levels (64). Additionally, PNX has been shown to elevate estrogen levels (53) and this suggests its mechanism of action may depend on the sex of the organism. Therefore, future studies must specify the sex of the organism model being used to elucidate the differential effects of PNX based on sex, which has often been neglected.

Most of the studies reviewed here that highlight PNX expression have been conducted predominantly in animal models. There is a notable paucity of research investigating PNX expression in human subjects or human-derived samples. This gap underscores the need for further exploration into the mechanisms underlying the actions of PNX to fully comprehend its physiological roles and potential therapeutic benefits. To translate findings from animal models into clinical practice, additional research is crucial to elucidate how PNX operates at the molecular level and how its modulation could impact human metabolic disorders. Such investigations are essential for identifying the therapeutic potential of PNX and developing targeted treatments for conditions where metabolic dysregulation plays a key role.

### 4.2 PNX and glucose metabolism

The involvement of PNX-14 in regulating insulin expression and secretion from pancreatic β cells suggests a critical regulatory function in managing glucose homeostasis and treating conditions like type 2 diabetes (33). The expression of PNX in pancreatic cells is induced by elevated glucose levels. A recent review has identified that the *Pnx* or *SMIM20* gene contains several transcription factor binding sites. Notable putative sites within the promoter of this gene include those for activating transcription factor (ATF) from the CREB family, peroxisome proliferator-activated receptor (PPAR) and retinoid X receptor (RXR) heterodimer, glucocorticoid receptor (GR), nuclear factor kappa B (NF-κB), estrogen receptor (ER), CCAAT/enhancer-binding protein (C/EBP), and octamer-binding transcription factor 1 (OCT1) (10). It is postulated that these transcription factors are influenced by high glucose levels, which enhance or suppress their activity, leading to the regulation of genes involved in metabolism, energy balance, stress response, and inflammation, as well as the expression of the *Pnx* or *SMIM20* gene. This complex regulatory network ensures proper cellular adaptation to elevated glucose levels, maintaining metabolic homeostasis.

The presence of PNX receptors and regulatory elements in the liver affects the expression of genes involved in glucose transport and metabolism, insulin-like growth factor expression, and ATP production (34). This influence alters glucose processing, ATP production, and gene expression related to glucose transport, potentially affecting liver function and glucose metabolism. These findings are supported by previous research showing that the activation of the cAMP/PKA pathway leads to the phosphorylation of CREB, which subsequently increases the expression of GLUT responsible for glucose uptake (65). Therefore, it is postulated that PNX may improve insulin resistance in diabetic patients by modulating gene expression in the pancreas and enhancing glucose uptake in the liver. Further studies in mammalian settings would strengthen this postulation as the current findings are demonstrated in a non-mammalian model.

Specifically, PNX-14’s regulation of insulin expression and secretion from pancreatic beta cells suggests a role in managing glucose homeostasis and treating conditions like type 2 diabetes (33). Its ability to alleviate NAFLD and exhibit cardioprotective effects during ischemia/reperfusion injury highlights its therapeutic potential in addressing liver and cardiac disorders associated with metabolic dysfunction (31, 60). Additionally, PNX-14’s promise in improving obesity-induced infertility by modulating mitochondrial dynamics underscores its potential role in reproductive health issues linked to metabolic disorders (53).

### 4.3 PNX and lipid metabolism

Lipid metabolism involves the synthesis and degradation of lipids in cells, including the breakdown or storage of fats for energy and the synthesis of structural and functional lipids. It consists of a balance between the catabolism and anabolism of lipids. Dysregulation of lipid metabolism can lead to metabolic disorders such as obesity, diabetes, and cardiovascular diseases (66). The recent findings have indicated the emerging role of PNX in modulating lipid metabolism.

As mentioned before, the promoter of *Pnx* or *SMIM20* gene consists of putative binding sites for several transcription factors known to regulate lipid metabolism (10). Transcription factors such as NF-κB, PPAR-RXR and C/EBP have been identified as key regulators of lipid metabolic pathways (67–70). Therefore, it is suggested that the expression of PNX is directly influenced by these transcription factors, thereby linking the regulatory role of PNX to lipid metabolism. Recent findings have supported this hypothesis, showing that fatty acids such as palmitic acid, docosahexaenoic acid and oleatecan modulate the expression of PNX (43).

Previous study has demonstrated that PNX promotes the differentiation of preadipocytes into mature adipocytes through the cAMP/Epac-dependent pathway (25). The activation of Epac influences various downstream signaling molecules and pathways, leading to adipogenesis (71, 72). By enhancing the differentiation of adipocytes, PNX helps increase the number of fat cells capable of storing lipids, thereby playing a role in lipid homeostasis. This function is particularly relevant in the context of obesity, where proper adipocyte differentiation and function can mitigate the adverse effects of excess lipid accumulation (73). Additionally, PNX has been shown to have protective effects against high-fat diet-induced NAFLD in mice (31). NAFLD is characterized by excessive fat accumulation in the liver, often due to dysregulated lipid metabolism, inflammation, and oxidative stress. (74). Previous studies demonstrated that PNX ameliorate the inflammation and oxidative stress (75, 76). Thus, it is suggested that PNX has protective effects prevent the pathological fat accumulation and could lead to novel therapeutic approaches for treating lipid metabolism disorders.

In non-mammalian vertebrates, PNX facilitates adipocyte differentiation via the Epac-ERK signaling pathway (44). The ERK pathway is well-known for its role in cell growth and differentiation as well as lipid metabolism (77). Furthermore, previous study has shown that the ERK is found to modulate the adipogenesis process (78). Further study is required to elucidate mechanism on how PNX regulates ERK pathways in details. The conservation of this mechanism across species highlights the evolutionary importance of PNX in lipid metabolism and suggests its potential as a therapeutic target not only in humans but also in other animals. Furthermore, PNX has the ability to mitigate endothelial cell dysfunction and regulate hyperphagia. The ability of PNX to regulate lipid metabolism through these conserved pathways underscores its versatility and significance as potential therapeutic applications in maintaining metabolic health conditions such as obesity.

### 4.4 PNX and electrolyte metabolism

Electrolytes are essential for maintaining cellular function, fluid balance, and overall homeostasis, and any imbalance can lead to significant health issues (79, 80). Notably, PNX is expressed in the hypothalamic region such as PVN and SON that secretes vasopressin and oxytocin (9, 81).Furthermore, previous finding of PNX has shown to stimulates vasopressin secretion (46). Therefore, PNX is suggested to have potential therapeutic applications in managing conditions related to fluid and electrolyte imbalance, such as dehydration and hyponatremia. (46, 47).

Beyond its central actions, PNX is also expressed in peripheral tissues such as the kidneys, which are vital for electrolyte regulation (12). Interestingly, the GPR173 also has found expressed in the kidney tissue (82). The kidneys organ is responsible to filter blood, reabsorb essential electrolytes, and excrete waste products, thus maintaining electrolyte balance (83). The presence of PNX and its receptor may influence renal function by modulating the activity of various ion channels and transporters involved in electrolyte reabsorption and excretion. This regulation is crucial for maintaining the balance of sodium, potassium, calcium, and other vital electrolytes. Conditions such as obesity, hypertension, and type 2 diabetes mellitus (T2DM) are often associated with disruptions in electrolyte metabolism (84–86). Understanding the mechanisms by which PNX regulates electrolyte balance is particularly relevant for therapeutic studies in the context of metabolic disorders.

### 4.5 PNX and food intake regulation

Food intake is essential for metabolism as it provides the calories and nutrients needed for energy production, growth, and maintenance of bodily functions (87). Carbohydrates, proteins, and fats from food are metabolized to produce energy, regulate blood glucose levels, and synthesize important biomolecules. Imbalances in food intake can disrupt these metabolic processes, leading to disorders such as obesity and diabetes (88–90).

PNX plays a multifaceted role in the regulation of food intake, primarily through its actions within the hypothalamus (91). Previous study has demonstrated that PNX increases food intake during the day while suppressing it at night (92). This finding suggests that PNX plays a role in regulating diurnal patterns of appetite, potentially influencing eating behavior based on the time of day. Recent reviews suggest that personalizing mealtimes may reduce circadian misalignment, enhance overall health, and help prevent metabolic disorder (93). By promoting daytime food intake and curbing nocturnal consumption, PNX may help align eating patterns with the natural circadian rhythms of the body, which could be beneficial for maintaining energy balance and metabolic health. Furthermore, the regulation of food intake by PNX is mediated through interactions with NPY1/NPY5 and CRF1/CRF2 receptors (6). Interestingly, CRF1 and CRF2 receptors are associated with the stress response and energy balance (94, 95). This dual mechanism suggests that PNX influences food intake by engaging both orexigenic neuropeptide pathways and stress-related signaling systems.

In infertile women with PCOS undergoing ovarian stimulation, higher baseline levels of nesfatin-1 (NES-1) and oxytocin (OT) were associated with pregnancy, while post-stimulation elevated phoenixin (PNX-14) levels predicted pregnancy. Pregnant women also showed increased post-stimulation levels of PNX-14, NES-1, and dopamine (DA) but decreased OT levels compared to non-pregnant counterparts. Additionally, a negative association between NES-1 and PNX was observed in pregnant participants, indicating potential roles for these neuroendocrine factors in reproductive success and their connection to metabolism warrants further investigation (50). Similarly, another study highlights their potential importance in regulating autonomic functions critical for maintaining metabolic homeostasis. With both NES-1 and PNX showing similar brain distributions, their co-expression in key hypothalamic nuclei suggests a potential role in metabolic regulation. Given the hypothalamus’s central role in integrating metabolic signals and regulating the hypothalamic-pituitary-gonadal axis, the wide distribution of PNX in conjunction with NES-1 suggests a possible involvement in metabolic pathways influencing reproductive health. Understanding the interplay between these neuropeptides and metabolic processes could provide insights into their roles in both metabolic regulation and reproductive function, warranting further investigation in contemporary neuroscience (49).

### 4.6 PNX and mitochondrial dynamics

Mitochondrial dynamics is comprising processes such as mitochondrial biogenesis, fission, fusion, and mitophagy (96). These processes are essential for maintaining cellular health and function. PNX has been shown to influence mitochondrial biogenesis through its interaction with key signaling pathways, including the cAMP/PKA and Epac-ERK pathways (107). By modulating these pathways, PNX can enhance the production of mitochondria, which is particularly important in tissues with high energy demands, such as muscle and brown adipose tissue (97). Furthermore, the promotion of neuronal mitochondrial biogenesis by PNX indicates potential neuroprotective effects and utility in managing neurodegenerative diseases associated with mitochondrial dysfunction (107).

Another study has explored the impact of phoenixin on obesity-induced infertility in female rats (53). The study revealed that PNX treatment significantly ameliorated several markers of metabolic and reproductive dysfunction. Specifically, PNX increased the expression of mitofusin 2 (Mfn2), electron transport chain (ETC) complex-I and mitochondrial transmembrane potential. These changes were accompanied by improvements in ovarian histopathology, decreased serum levels of insulin and testosterone, as well as ovarian levels of dynamin-related protein 1 (Drp1), reactive oxygen species (ROS), TNF-α, MDA, and caspase-3. Therefore, it is suggested that PNX can counteract the adverse effects of obesity on fertility by modulating mitochondrial dynamics and reducing oxidative stress. Collectively, these insights pave the way for further exploration of phoenixin as a therapeutic agent in metabolic, fertility, and neurodegenerative disorders.

### 4.7 Integration with reproductive functions

Metabolism and reproduction are integrated through a complex, multifaceted process (98). This integration ensures that reproductive success is closely aligned with the metabolic state of an organism. PNX has emerged as a significant mediator in this interplay, influencing both metabolic and reproductive functions (9). Metabolic hormones like insulin and leptin are known to influence reproductive hormone levels (99, 100). PNX modulates the activity of these metabolic hormones, thereby indirectly affecting reproductive hormones such as GnRH and LH (12). This hormonal interplay ensures that reproductive activities are aligned with the metabolic state of the organism (101).

Reproductive processes are energy-intensive and demand a well-regulated energy balance (102). PNX contributes to this balance by interacting with hormones that regulate appetite and energy expenditure, ensuring adequate energy availability for reproductive activities (33, 38, 45). Furthermore, PNX plays a significant role in mitochondrial dynamics, encompassing biogenesis, fission, fusion, and mitophagy (53, 107). These processes are vital for maintaining mitochondrial health and energy production, which are crucial for both metabolic and reproductive health (103, 104). Efficient mitochondrial function supports the energy needs of cellular processes and the synthesis of hormones and signaling molecules essential for reproduction.

Understanding the integrative role of PNX in metabolism and reproduction opens new avenues for therapeutic interventions. One pertinent example is PCOS, a disorder characterized by metabolic and reproductive dysfunctions, including insulin resistance, obesity, and irregular menstrual cycles (105). Studies have shown that PNX influences insulin sensitivity and glucose uptake, which are crucial factors in managing insulin resistance in PCOS (33). By modulating these metabolic pathways, PNX has the potential to alleviate some of the metabolic burdens associated with PCOS. Additionally, the role of PNX in lipid metabolism, including the regulation of lipogenesis and lipolysis (25, 31), could help manage obesity and its related complications in PCOS patients. Given the intriguing potential role of PNX in regulating reproduction and metabolism, a recent study demonstrated that knocking out the PNX gene did not affect fertility (45). This observation may be attributed to compensatory mechanisms that ensure the survival and continuity of the organism (106). Thus, warrant further study to elucidate the role of PNX in fertility.

Given the dual role of PNX in regulating both metabolic and reproductive functions, it presents a unique therapeutic target for managing PCOS. Further research is essential to fully elucidate the mechanisms through which PNX exerts its effects on metabolic and reproductive pathways in PCOS. Clinical studies exploring PNX levels in PCOS patients and the impact of PNX-based therapies on metabolic and reproductive outcomes are particularly warranted.

## 5 Future direction

Most of the studies reviewed have been conducted in animal models, including rats and zebrafish, and *in vitro* cell cultures. While these models provide valuable insights into the mechanisms of PNX action, the relevance of these findings to human physiology remains an open question. Future research should focus on validating these findings in human tissues and clinical studies to ascertain the translational potential of PNX as a therapeutic target. Meanwhile, the precise mechanisms through which PNX exerts its effects are not fully understood. Both PNX-14 and PNX-20 are known to interact with the G protein-coupled receptor 173 (GPR173), but the downstream signaling pathways and receptor interactions need further elucidation. Understanding the specific pathways activated by PNX, such as the cAMP/PKA pathway and CREB phosphorylation, is crucial for developing targeted therapeutic interventions. Additional mechanistic studies are needed to delineate how PNX modulates these pathways in different tissues and under various physiological and pathological conditions.

The actions of PNX appear to be context-dependent, with some studies reporting contradictory findings. For instance, while PNX-14 has been shown to enhance insulin secretion and protect against NAFLD, other studies might not observe these effects under different experimental conditions. This variability suggests that PNX’s effects might be influenced by factors such as dosage, the specific isoform used (PNX-14 vs. PNX-20), and the physiological state of the organism. Future studies should aim to standardize experimental conditions and dosages to better compare results across different studies. In addition, PNX’s role in the neuroendocrine regulation of metabolism is particularly intriguing. The peptide’s ability to influence reproductive hormones, such as LH and FSH, and its involvement in food intake and stress responses point to a broader role in maintaining metabolic homeostasis. PNX’s interaction with the GnRH system and its impact on neuroendocrine functions suggest that it could serve as a bridge linking metabolic and reproductive health.

Further research should explore these neuroendocrine interactions in more detail to understand how PNX contributes to overall metabolic regulation and its potential implications for disorders such as PCOS and T2DM. The potential prognostic significance of PNX in metabolic disorders needs to be investigated further. Elevated levels of PNX-20 in individuals with type 2 diabetes and its association with diabetic complications suggest that PNX could serve as a biomarker for disease progression. Studies should aim to establish clear correlations between PNX levels and clinical outcomes in metabolic disorders. This could involve longitudinal studies tracking PNX levels in patients over time to determine their predictive value for disease onset and progression. The diverse effects of PNX-14 and PNX-20 on insulin secretion, liver function, cardiovascular health, and adipogenesis highlight their potential as therapeutic targets. Developing PNX-based therapies could involve designing peptide analogs or small molecules that modulate PNX signaling. Such therapies could be aimed at enhancing insulin secretion in diabetes, protecting against liver and cardiac injuries, or improving reproductive health in conditions like obesity-induced infertility. Clinical trials will be necessary to evaluate the efficacy and safety of these potential treatments.

Despite these advancements, several limitations remain. Most studies focus on animal models, raising questions about translatability to human physiology. Variability in experimental designs and methodologies complicates data synthesis and interpretation. Additionally, while many studies highlight PNX’s beneficial effects, some contradictory findings suggest context-dependent actions that require further elucidation. Understanding PNX signaling mechanisms and receptor interactions remains incomplete, necessitating additional research to delineate its mode of action in metabolism.

## 6 Conclusion

In conclusion, PNX peptides, particularly PNX-14 and PNX-20, exhibit a wide range of effects on metabolic processes, hormonal regulation, and cellular function. While the current body of research provides a strong foundation, many questions remain regarding the mechanisms of PNX action, its relevance to human health, and its potential as a therapeutic target. Addressing these questions through rigorous mechanistic studies, translational research, and clinical trials will be crucial for harnessing the therapeutic potential of PNX in metabolic disorders and related conditions. The prognostic significance of PNX in the wider neuroendocrine scenario also warrants further investigation, offering potential insights into the role of PNX in metabolic homeostasis and disease.
